# Calendar

**DOI:** 10.1155/1995/13862

**Published:** 1995


					Calendar
Date Venue Event Contact Address
November Strasbourg, European Institute ofTele- Secretariat du Prof. Jaques Mar
24-25 France surgery: Advanced Course in IRCAD, Hospital Civil, Strasbourg Cedex 67092
LaparoscopicHepatobiliary France
and Pancreatic Surgery Phone: +33 88 11 90 00 Fax: +33 88 11 90 99
December Hong Kong 7th Wilson S Wang
1-3 Hong Kong International Surgical
Symposium Frontiers
in Surgery
Symposium Secretariat, 7th Wilson S Wang
International Surgical Symposium,
Department of Surgery, Prince ofWales
Hospital, Shatin, New Territories, Hong Kong
Phone: (852) 2632 2632 Fax: (852) 2645 3602
December Hong Kong 10th International Workshop Sydney Chung, Endoscopy Centre,
5-7 Hong Kong on Therapeutic Endoscopy; Prince ofWales Hospital,
10th International Meeting on The Chinese University of Hong Kong,
Therapeutic Endoscopy Shatin N.T., Hong Kong
Phone: (852) 2632 2233 Fax: (852) 2632 0075
1996
March
20-22
Brighton
UK
British Society for Gastro- Mr Colin Johnson, University Surgical Unit,
enterology: Pancreatic Section Tremona Road, Southampton SO9 4XY,
International Symposium UK

				

